# Point of care testing in Croatia: a survey of the Working group for point of care testing of the Croatian society of medical biochemistry and laboratory medicine

**DOI:** 10.11613/BM.2024.010703

**Published:** 2023-12-15

**Authors:** Ivana Baršić Lapić, Lara Milevoj Kopčinović, Nedjeljka Ruljančić, Marija Grdić Rajković, Maja Kuštro

**Affiliations:** 1 Department for Laboratory Diagnostics, University Hospital Center Zagreb, Zagreb, Croatia; 2 Department of Clinical Chemistry, Sestre Milosrdnice University Hospital Center, Zagreb, Croatia; 3 Medical Biochemistry Laboratory, University Psychiatric Hospital Sveti Ivan, Zagreb, Croatia; 4 Faculty of Dental Medicine and Health, Josip Juraj Strossmayer University of Osijek, Osijek, Croatia; 5 Department of Medical Biochemistry and Hematology, Faculty of Pharmacy and Biochemistry, University of Zagreb, Zagreb, Croatia; 6 Department of Laboratory Diagnostics, General Hospital Dubrovnik, Dubrovnik, Croatia

**Keywords:** point of care, questionnaires, quality control, standardization

## Abstract

**Introduction:**

The aim of this study was to investigate attitudes and routine procedures in point of care testing (POCT) among non-laboratory and laboratory healthcare professionals in Croatia.

**Materials and methods:**

The Working Group (WG) for POCT of the Croatian society of medical biochemistry and laboratory medicine has designed two anonymous surveys for laboratory staff and non-laboratory staff with a total of 44 questions/statements on POCT (27 questions for non-laboratory staff and 17 for laboratory staff). Surveys were sent to 184 medical biochemistry laboratory (MBL) managers, the Croatian medical chamber and the Croatian chamber of nurses. The survey was disseminated using the online survey platform SurveyMonkey.

**Results:**

A total of 112 non-laboratory healthcare professionals and 50 laboratories participated in the survey, which represents a response rate of 0.25% for non-laboratory professionals and 27% for MBLs. The majority of non-laboratory staff stated that POCT enables better medical care for the patient (90/112) and that the implementation of new POCT devices should be the responsibility of a POCT team comprising laboratory and clinical healthcare professionals. The great majority of responding MBLs (42/50) acknowledge that POCT is necessary for better patient care, and also realize that validation of POCT devices and comparison to the central laboratory is necessary before implementation (49/50).

**Conclusions:**

The majority of participants consider POCT as a medical tool that enables better patient care but there is still a lack of communication between laboratory and clinical staff. The study identified some critical spots that will help to create national guidelines to ensure high patient safety when using POCT devices.

## Graphical abstract

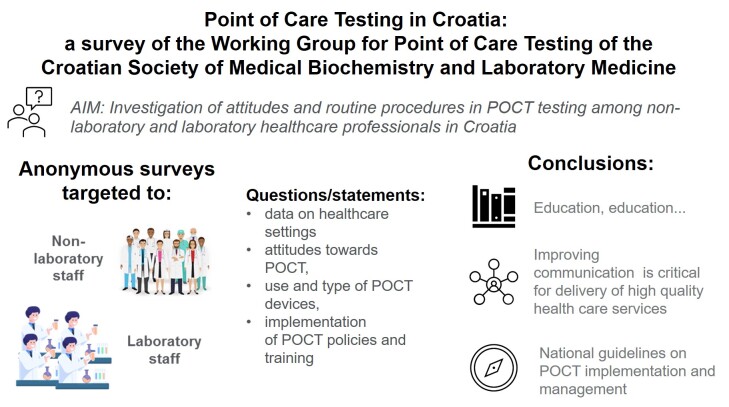

## Highlights

Majority of participants find point of care testing a valuable tool that can enhance patient care

Communication between laboratory and clinical personnel should be improved

National point of care testing regulations are available but not implemented

Education and training are crucial in ensuring high-quality results and patient safety

## Introduction

Point-of-care testing (POCT) is defined as testing performed in the proximity of patient care, outside the central laboratory environment (*1*-*5*). This allows for more expeditious clinical decisions and speeds up patient management. The unprecedented advances in technology and miniaturization, along with the shorter turnaround time (TAT) of POCT results have led to an increased demand for POCT in all healthcare settings which is witnessed by the exponential growth of the POCT market worldwide (*6*-*8*).

Over time, the number and type of analyses performed near the patient’s bedside have increased due to its many advantages. Besides the shortened TAT, decreased iatrogenic blood loss, rapid response to critical results, and decreased therapeutic TAT are some of the main reasons why the implementation of POCT devices should be considered. However, the benefits of implementing POCT must be carefully weighed against its limitations.

Point-of-care testing is usually performed by non-laboratory staff, emphasizing the need to organize, evaluate and monitor training and competence for all personnel performing POCT (*2*, *5*). Education should be an ongoing process and must include theoretical and practical information (*5*). Since POCT is performed outside the central laboratory, it is very important to establish a management system (including quality control, consumption of reagents, critical results, duplicate samples, competence evaluation, documentation control *etc*.) supervised by a body including laboratory professionals (*4*, *5*). To ensure good quality, all personnel should be actively involved in POCT implementation (*5*).

Preanalytical errors are the most common in POCT and include incorrect timing of sample collection, errors in blood collection, errors in patient identification, errors in sample collection, underfilled tubes, inadequate mixing of samples and hemolyzed samples that can cause false results (*4*, *9*, *10*). Failure to detect them can cause wrong medical decisions and negatively affect patient safety. Today there is a growing awareness that the majority of problems that arise with POCT are due to incorrect implementation of POCT devices. These problems are due to incorrect sampling techniques, poor operator experience and training, inappropriate interpretation of results and the absence of appropriate quality control procedures (*4*). All these problems can lead to unreliable results that may have serious implications for patients.

There are several international guidance documents for POCT that focus on quality management, selection of point of care testing devices based on patient care and clinical needs, and guidance for users of *in vitro* diagnostic devices outside the central laboratory to ensure reliable results comparable to those from central laboratories (*5*, *11*, *12*). Similarly, in recognition of the growing interest in POCT in Croatia, the Croatian chamber of medical biochemists (CCMB) recognizes POCT as an integral part of laboratory diagnostics. Therefore, the Croatian ministry of health issued two national regulations concerning POCT diagnostics in 2005 and 2019. These regulations were issued in collaboration with the CCMB and the Croatian medical chamber (CMC). According to both documents, POCT must be performed under the supervision of a central laboratory.

In 2015 the Croatian society of medical biochemistry and laboratory medicine (CSMBLM) established a Working group for point of care testing (WG POCT) whose main aim is to propose national standards for POCT implementation, in and outside the hospital setting. Our hypothesis is that there is little or no control for POCT at the national level despite existing regulations. Therefore, we conducted a survey among non-laboratory and laboratory staff to review current attitudes and practices regarding POCT in Croatia. The aim was to identify the weakest points and find solutions for improvement.

## Materials and methods

### Study design

The study was conducted using two anonymous surveys written by members of the CSMBLM’s WG POCT and targeted to laboratory professionals and clinical staff in general. It included 27 questions/statements for non-laboratory professionals and 17 questions/statements for laboratory professionals related to POCT attitudes, policies and procedures. The survey was conducted from November 2015 to February 2016. It was sent to 184 MBL managers, CMC and the Croatian chamber of nurses (CCN). Laboratory managers were identified using the CSMBLM database. A web link to the questionnaire was sent directly to CMC and CCN with a request to the chambers to inform and ask their members to participate in the questionnaire. The survey was disseminated using the online survey platform SurveyMonkey (SurveyMonkey Inc., Palo Alto, USA). Participants were guaranteed anonymity in the survey.

The questions/statements were divided into five groups to obtain specific data on healthcare settings, attitudes towards POCT, use and type of POCT devices, and implemented POCT policies and training.

### Statistical analysis

The results are presented as numbers and percentages (if N ≥ 100) or numbers and proportions (if N < 100). The SurveyMonkey online survey platform was used for data collection and calculation.

## Results

A total of 112 non-laboratory healthcare professionals and 50 laboratories participated in the survey, which corresponds to a response rate of 0.25% for non-laboratory healthcare professionals and 27% for MBLs (according to the Croatian health statistics yearbook 2015 there were a total of 44,142 healthcare workers: nurses and physicians). The general characteristics of the participants are presented in Table 1 and attitudes and routine practices related to POCT for non-laboratory healthcare professionals are presented in Table 2. In addition to the basic POCT questions, the survey for non-laboratory and laboratory professionals included questions about the use of POCT devices in routine work, so not all participants answered all questions. Table 2 and 3 also show the number of participants for each question.

**Table 1:** General characteristics of participants

	**Answer, N (%) or N (ratio)**	
**Statement**	**Non-laboratory healthcare professionals** **(N = 112)**	**Laboratory professionals** **(N = 50)**
Please state your profession.		
Physician	88 (78)	/
Nurse	24 (21)	
Please indicate your employment institution.		
University hospital centre	30 (27)	9 (0.18)
Clinic (clinical hospital)	14 (12)	11 (0.22)
General hospital	9 (8)	10 (0.20)
Primary healthcare	27 (24)	18 (0.36)
Private institution/clinic	11 (10)	2 (0.04)
Emergency medicine services	20 (18)	/
Other (nonspecified)	1 (1)	/
Please state your work experience.		
< 1 year	1 (1)	/
1-5 years	17 (15)	
5-10 years	17 (15)	
> 10 years	77 (69)	

**Table 2:** Answers provided by non-laboratory healthcare professionals

**Question/statement (N of participants)**	**Answer,** **N (%) or N (ratio)**
1. In your opinion, POCT refers to: (N = 112)	
emergency (STAT) tests	42 (38)
any laboratory/diagnostic test	65 (58)
I do not know	5 (4)
2. In your opinion, POCT*: (N = 112)	
is not necessary.	2 (2)
enables rapid results reception.	77 (69)
enables better patient care.	90 (80)
is a burden for clinical staff.	14 (13)
is expensive.	13 (12)
3. Implementation of new POCT devices and their organization should be the responsibility of: (N = 112)	
laboratory staff.	1 (1)
clinical staff.	11 (9)
a team composed of laboratory and clinical staff.	100 (89)
4. In your routine practice, do you use POCT devices or results obtained from POCT devices? (N = 112)	
Yes.	90 (80)
No.	22 (20)
5. List the POCT devices that you use. (N = 81)	
Glucometers	73
Blood gas analysers	11
PT/INR device	6
HbA1c analysers	4
Haematology analysers (+CRP)	1
Aggregometry device	1
Benchtop critical care analyser (cardiac panel)	1
6. How long have you used POCT devices? (N = 84)	
For < 1 year.	5 (0.06)
For 1-5 years.	26 (0.31)
For 5-10 years.	12 (0.14)
More than 10 years.	41 (0.49)
7. POCT devices are: (N = 86)	
easy to use.	64 (0.74)
complicated to use.	0 (0)
ease-of-use depends on the device.	22 (0.26)
8. Have you been trained for working on the POCT device? (N = 90)	
Yes.	29 (0.32)
No.	59 (0.66)
No education is needed for POCT devices.	2 (0.02)
9. Who was responsible for your POCT training?* (N = 88)	
Laboratory personnel.	7 (0.08)
Supplier/manufacturer representatives.	32 (0.36)
A colleague provided the necessary information for POCT handling.	19 (0.22)
No education was performed.	40 (0.45)
10. In your opinion, is it necessary to periodically perform re-education for POCT devices? (N = 89)	
Yes.	53 (0.60)
No, one education cycle is enough.	25 (0.28)
I don’t know.	11 (0.12)
11. Is internal quality control using control materials performed on POCT devices? (N = 87)	
Yes.	39 (0.45)
No.	25 (0.29)
I don’t know.	23 (0.26)
12. If the answer to the previous question is Yes, please state who performs internal quality control rocedures? (N = 38)	
Laboratory personnel.	15 (0.39)
Supplier/manufacturer representatives.	5 (0.14)
Nurses/physicians.	18 (0.47)
13. How frequently do you use POCT devices/results in your routine practice? (N = 83)	
Daily.	50 (0.60)
A few times a week.	26 (0.31)
Once a month.	5 (0.06)
Once every several months.	2 (0.03)
14. How frequently are POCT results helpful in faster decision making pertaining to patient management? (N = 88)	
Always.	49 (0.56)
Sometimes.	29 (0.33)
Never.	0 (0)
Not applicable.	10 (0.11)
15. In your opinion, did POCT results shorten the length of stay of patients in your institution? (N = 87)	
Always.	13 (0.15)
Sometimes.	46 (0.53)
Never.	12 (0.14)
I don’t know.	16 (0.18)
16. If you work in primary or private health care institution, have POCT results influenced your decision not to send the patient to the emergency department? (N = 59)	
Yes.	37 (0.63)
No.	22 (0.37)
17. Have POCT results helped fasten decision making regarding therapy management? (N = 80)	
Always.	21 (0.26)
Sometimes.	50 (0.63)
Never.	4 (0.05)
I don’t know.	5 (0.06)
18. Have POCT devices helped reduce the number of requests to medical biochemistry laboratories? (N = 84)	
Yes.	43 (0.51)
Sometimes.	29 (0.35)
Never.	7 (0.08)
I don’t know.	5 (0.06)
19. Are POCT devices used only for urgent requests (results needed within 30 minutes)? (N = 85)	
Yes.	47 (0.55)
No, they are used regardless of urgency.	38 (0.45)
20. Do you find POCT devices useful? (N = 85)	
Yes.	78 (0.92)
No.	3 (0.03)
I don’t know.	4 (0.05)
21. How often do you send patient samples to medical-biochemistry laboratories for retesting due to uncertainty of results obtained with POCT devices? (N = 76)	
Every POCT result is sent for retesting to medical-biochemistry laboratories regardless of the results obtained.	16 (0.21)
Sometimes.	60 (0.79)
22. How often do you review POCT results in written form (in printout or as electronic record)? (N = 83)	
Always.	51 (0.61)
Only for some patients.	23 (0.28)
Never, results are communicated verbally.	9 (0.11)
23. Are POCT results documented/stored in the patient’s medical record? (N = 84)	
Always.	76 (0.90)
Sometimes.	3 (0.04)
Never.	5 (0.06)
24. When issues/technical problems arise with POCT devices, who is notified?* (N = 83)	
Laboratory personnel.	18 (0.22)
Supplier/manufacturer representatives.	61 (0.73)
Others (superior staff, procurement service, *etc*.)	16 (0.19)
*Multiple choices available. POCT - point of care testing.	

**Table 3:** Answers provided by laboratory healthcare professionals

**Question/statement (N of participants)**	**Answer (ratio)**
1. Are POCT tests performed in your institution? (N = 50)	
Yes.	29 (0.58)
No.	13 (0.26)
I do not know.	8 (0.16)
2. List the POCT devices used in your institution. (N = 50)	
Blood gas analysers	14
Glucometers	19
HbA1c analysers	2
Haematological analysers (+ CRP)	2
Platelet aggregometer	1
Benchtop critical care analyser (cardiac panel)	1
3. Is the laboratory involved in POCT devices implementation? (N = 29)	
Yes.	18 (0.62)
No.	11 (0.38)
4. Is there a designated POCT team in your institution responsible for all POCT devices? (N = 29)	
Yes.	16 (0.55)
No.	13 (0.45)
5. Is there an institutional written policy (document) pertaining to POCT? (N = 29)	
Yes.	14 (0.48)
No.	15 (0.52)
6. Is training for the POCT devices summarized in a written educational plan? (N = 29)	
Yes.	14 (0.48)
No.	15 (0.52)
7. Are POCT training records documented and stored? (N = 27)	
Yes.	15 (0.56)
No.	12 (0.44)
8. Is internal quality control performed on POCT devices? (N = 28)	
Yes.	23 (0.82)
No.	5 (0.18)
9. Is external quality assessment performed on POCT devices? (N = 29)	
Yes.	18 (0.62)
No.	11 (0.38)
10. In your opinion, POCT*: (N = 50)	
is not necessary.	5 (0.10)
is necessary for better patient care.	42 (0.84)
is an additional burden for laboratory staff.	17 (0.34)
is an additional burden for clinical staff.	9 (0.18)
11. Before implementation it is necessary to validate POCT results and compare them to those from the laboratory. (N = 50)	
Yes.	49 (0.98)
No.	1 (0.02)
12. Do you think that the laboratory should participate in the organization and implementation of POCT? (N = 50)	
Yes.	47 (0.94)
No.	3 (0.06)
13. Are you familiar with the regulations of the Croatian Chamber of Medical Biochemists on POCT? (N = 50)	
Yes.	44 (0.88)
No.	6 (0.02)
*Multiple choices available. POCT - point of care testing.	

The majority of non-laboratory professionals stated that POCT enables better medical care for the patient (90/112) and rapid test results receipt (77/112). However, a few of them, declared that POCT is expensive and represents an additional burden to their routine work. More than half (65/112) of the responding non-laboratory healthcare professionals stated that, in their opinion, POCT testing comprises any laboratory/diagnostic test. Most of the participants (100/112) in this group declared that the implementation of new POCT devices, policies, and practices should be the responsibility of a POCT team comprising laboratory and clinical healthcare professionals. The majority of participants (90/112) confirmed the use of POCT devices/results in their routine practice. The most commonly used devices declared by participants are listed in Table 2. The majority of non-laboratory healthcare professionals using POCT declared a faster patient management process, shorter length of stay, and reduced number of samples sent to the central laboratory for testing. However, 0.79 (60/76) of respondents stated that they send an additional sample to the laboratory for retesting in case of an uncertain POCT result: most often in case of discrepancies between POCT results and clinical presentation, critical or unreliable POCT results. The majority of respondents (76/84) declared that POCT results are always stored in the patient’s medical record.

Table 3 presents POCT practices for laboratory healthcare professionals responding to our survey. Blood gas analyzers and glucometers are the most commonly used POCT devices. Over half (18/29) of participating MBLs declared that the laboratory is involved in POCT implementation with a designated POCT team organized in 0.53 (16/29) of participating MBLs. About half of responding MBLs stated that no written institutional policy related to POCT and no training plan is available in their institutions. The majority of respondents stated that laboratory staff was responsible for POCT training and 15/27 declared that POCT training is documented. Internal quality control and external quality assessment procedures for POCT devices have been instituted in 23/28 and 18/29 responding institutions, respectively.

## Discussion

The development of a range of POCT devices that provide clinicians with laboratory test results more quickly could provide an opportunity to improve the quality of patient care. Since POCT involves tests conducted outside of a laboratory setting, it is crucial to focus on the implementation process and ensure consistently high quality at every step. In this study, we aimed to investigate attitudes and routine procedures in POCT testing among non-laboratory and laboratory healthcare professionals in Croatia. Our results show that laboratory professionals are aware of the national POCT regulations but in general, not all of them are actively involved in POCT implementation and supervision. Another significant result is poor communication between non-laboratory and laboratory healthcare professionals in the POCT field. These results confirm our hypothesis on poor POCT control nationwide and call for immediate action in terms of applicable guidance documents.

The majority of participants, both laboratory and non-laboratory professionals, consider POCT as a medical tool that enables better patient care, primarily because of the rapid availability of test results. Similar results were obtained in a study among general practitioners in Germany, where the vast majority of participating German general practitioners (93%) rated POCT as a useful diagnostic tool in their practice (*13*). In our study the majority of non-laboratory healthcare professionals (0.66) stated that no education on the POCT device used was provided, while a great proportion of non-laboratory users declared that training was performed exclusively by POCT suppliers/manufacturers. If we consider the statement that POCT allows clinicians to make clinical decisions without sending patients to the emergency department and that they do not always repeat the analysis in the central laboratory, communication between non-laboratory and laboratory personnel is imperative to ensure patient safety.

Proper training of non-laboratory personnel in the use of POCT equipment can change the way patients are treated and have tremendous benefits for health care overall. Training must include the basic operating steps of a POCT instrument, information about sample preparation and common analytical interferences, recognizing unreliable results, comparing POCT results with results from the central laboratory-whether the methods are comparable, whether there are sample-specific differences between POCT and the central laboratory *etc*. (*5*). When conducting training, one should always keep in mind the lack of laboratory knowledge among clinical staff. This could be quite a challenge, as the use of POCT devices requires prior knowledge of preanalytical and analytical issues. This knowledge can better identify errors and prevent incorrect clinical decisions.

Hospitals have their own laboratories, but the situation is different in primary care because not every primary care physician has a laboratory nearby. The great benefit of POCT could be seen in primary health care as a first-line treatment for the patient. Our study included only a small number of primary care participants, but their comments about how POCT helped them avoid sending a patient to the emergency department are significant. Implementing POCT in primary care could provide economic benefits by reducing the burden on the emergency department. However, this should be done using standard practices, guidelines, and regulations. All parties involved in POCT should cooperate, *i.e*. suppliers/manufacturers on the one end and a POCT team made up of laboratory professionals and clinical staff on the other. Implementation is crucial for the safe use of POCT. Every participant’s responsibilities should be clearly outlined in this first step. Using POCT as an auxiliary medical tool without proper education is not only unsafe for the patient but also a financial burden on the healthcare system. Since this diagnostic is growing rapidly, more enthusiasts are needed to initiate the safe and cost-effective implementation of POCT diagnostics.

Our study shows that the laboratory staff is aware of what is mandatory for POCT but there is still a lack of communication with clinical staff. Laboratory professionals should explain how POCT should be used using a scientific and professional background. Clinical staff wants fast diagnostics that allow fast treatment introduction, reduction in hospital stays, and satisfied patients. It is therefore mandatory that these two professions work closely together to choose the right POCT device for the right patient. In addition, they must resolve unexpected results. This is to prevent more serious consequences for the patient due to an incorrect report. Point of care testing has become the standard in critical emergencies due to the immediate availability of critical results and rapid therapeutic turnaround time. The goal of POCT is to enable rapid response and improve patient outcomes, not to increase the incidence of medical errors. In the study from Kost, experts recommended validating trained and certified operators before using hospital-based instruments for POCT (*14*).

It should also be emphasized that our study has some limitations. Firstly, the low response rate of this nationwide survey hampers the generalization of the results. However, it must be noted that our results provide a fair understanding of Croatia’s POCT routine practice. Furthermore, our participants self-reported and could not be independently verified.

In conclusion, this study shows that non-laboratory and laboratory staff consider POCT as a tool for better patient care, and they know the need for training and performing quality control. Our study will help the WG for POCT create proper national guidelines for implementing and managing POCT.

## Data Availability

The data generated and analyzed in the presented study are available from the corresponding author upon reasonable request.

## References

[1] de Vries C Doggen C Hilbers E Verheij R IJzerman M Geertsma R Results of a survey among GP practices on how they manage patient safety aspects related to point-of-care testing in every day practice. BMC Fam Pract. 2015;16:9. 10.1186/s12875-014-0217-2 25648985 PMC4332919

[2] Shaw JLV. Practical challenges related to point of care testing. Pract Lab Med. 2015;4:22–9. 10.1016/j.plabm.2015.12.002 28856189 PMC5574506

[3] Price CP. Point of care testing. BMJ. 2001;322:1285–8. 10.1136/bmj.322.7297.1285 11375233 PMC1120384

[4] Giavarina D Villani A Caputo M. Quality in point of care testing. Biochem Med (Zagreb). 2010;20:200–6. 10.11613/BM.2010.024

[5] Clinical and Laboratory Standards Institute (CLSI). Essential Tools for Implementation and Management of a Point-of-Care Testing Program. 3rd ed. CLSI guideline POCT04. Wayne:CLSI;2016.

[6] Asha SE Chiu Fat Chan A Walter E Kelly PJ Morton RL Ajami A Impact from point-of-care devices on emergency department patient processing times compared with central laboratory testing of blood samples: a randomised controlled trial and cost-effectiveness analysis. Emerg Med J. 2014;31:714–9. 10.1136/emermed-2013-202632 23748157

[7] Khan AH Shakeel S Hooda K Siddiqui K Jafri L. Best practices in the implementation of a point of care testing program: experience from a tertiary care hospital in a developing country. EJIFCC. 2019;30:288–302. 31695586 PMC6803771

[8] Quinn AD Dixona D Meenan BJ. Barriers to hospital-based clinical adoption of point-of-care testing (POCT): A systematic narrative review. Crit Rev Clin Lab Sci. 2016;53:1–12. 10.3109/10408363.2015.1054984 26292075

[9] Ehrmeyer SS Laessig RH. Point-of-care testing, medical error, and patient safety: a 2007 assessment. Clin Chem Lab Med. 2007;45:766–73. 10.1515/CCLM.2007.164 17579530

[10] Plebani M. Does POCT reduce the risk of error in laboratory testing? Clin Chim Acta. 2009;404:59–64. 10.1016/j.cca.2009.03.014 19298804

[11] Clinical and Laboratory Standards Institute (CLSI). Quality Management: Approaches to Reducing Errors at the Point of Care; Approved Guideline. CLSI document POCT07-A. Wayne:CLSI;2010.

[12] Clinical and Laboratory Standards Institute (CLSI). Selection Criteria for Point-of-Care Testing Devices; Approved Guideline. CLSI document POCT09-A. Wayne: CLSI;2010.

[13] Matthes A Wolf F Schmiemann G Gágyor I Bleidorn J Markwart R. Point-of-care laboratory testing in primary care: utilization, limitations and perspectives of general practitioners in Germany. BMC Prim Care. 2023;24:96. 10.1186/s12875-023-02054-0 37038122 PMC10088261

[14] Kost GJ. Preventing medical errors in point-of-care testing: security, validation, safeguards, and connectivity. Arch Pathol Lab Med. 2001;125:1307–15. 10.5858/2001-125-1307-PMEIPO 11570905

